# Malignant Neoplasm of the Meninges: A 20-Year Retrospective Study to Assess Trends and Disparities in Place of Death

**DOI:** 10.7759/cureus.69424

**Published:** 2024-09-14

**Authors:** Anish Chandran Chandra Senan, Steffi John, Fardin Akbar Hyderi, Yves H Jean, Aarav Godavarthi, Rakshya Adhikari

**Affiliations:** 1 Internal Medicine, Dr. NTR University of Health Sciences, Vijayawada, IND; 2 Internal Medicine, Southwestern University PHINMA, Cebu, PHL; 3 Internal Medicine, Bangladesh Medical College, Bangladesh Medical Studies and Research Institute (BMSRI), Dhaka, BGD; 4 General Medicine, Universite Notre Dame D'Haiti, Port-au-Prince, HTI; 5 Internal Medicine, Shadow Creek High School Houston, Texas, USA; 6 Internal Medicine, Kathmandu Medical College, Kathmandu, NPL

**Keywords:** cdc wonder (wide-ranging online data for epidemiologic research) database, end of life care, home or hospice care, malignant neoplasm of meninges, mortality trends, palliative care

## Abstract

Background: The site of death is a crucial factor associated with the tumor's progression and complications arising from it; therefore, analyzing nationwide patterns in place of death is essential. The present paper aims to evaluate the disparities in place of death for malignant neoplasm of the meninges using the CDC-WONDER (Centers for Disease Control and Prevention Wide-ranging ONline Data for Epidemiologic Research) database over 22 years (1999-2020).

Methods: CDC-WONDER data from 1999 to 2020 were analyzed to investigate mortality trends related to malignant neoplasm of the meninges. Data selection ensured inclusivity of all races, with confidentiality and death count thresholds considered. Demographics encompassed Census Regions, all genders, races, and 10-year age groups, along with a five-year forecast. Microsoft Excel (Microsoft Corporation, Redmond, Washington) and R Software (R Foundation, Boston, Massachusetts) were used for data processing and statistical analysis, with visualization through ARIMA modeling.

Results: Cumulative home and hospice deaths were analyzed based on overall age, gender, race, and region, demonstrating that home and hospice deaths increased overall, particularly in the 65-74 and 75-84 age groups, and more so in females. White individuals showed increasing trends, while Black or African American individuals remained stable. Regionally, the South had the highest increase, while the Northeast remained stagnant.

Conclusion: There is a concerning upward trend in home or hospice deaths among individuals with malignant neoplasm of the meninges, particularly within the 65-84 age group, in females, among White individuals, and in the Southern region. More comprehensive data is needed, and further research must be conducted to understand the underlying causes for the rise in these demographics and to improve healthcare facilities.

## Introduction

Malignant neoplasms of the meninges, a group of rare and formidable brain tumors originating in the protective membranes surrounding the brain and spinal cord, pose a significant challenge to both patients and healthcare systems [1]. Despite their infrequent occurrence, these tumors exert a substantial toll on affected individuals, often leading to devastating outcomes. One crucial aspect of this battle against meningeal neoplasms is understanding where these patients ultimately face their end and the factors that influence this critical decision [2,3].

The cause of death in individuals with malignant neoplasm of the meninges is overwhelmingly associated with the tumor's progression and complications arising from it. The insidious growth of these tumors can lead to a myriad of neurological symptoms, including severe headaches, seizures, and cognitive impairments, which can ultimately result in neurological failure and death [2]. With a five-year survival rate of approximately 35.7%, these neoplasms underscore the importance of investigating not only the factors contributing to mortality but also where these patients experience their final moments [4].

This research article delves into the place of death disparities among individuals diagnosed with malignant neoplasm of the meninges in the United States over a span of 22 years. Utilizing data from the Centers for Disease Control and Prevention’s Wide-ranging Online Data for Epidemiologic Research (CDC-WONDER) database, we aim to elucidate the trends and determinants associated with where these patients breathe their last breath and shed light on whether geographic, demographic, or healthcare-related factors influence the place of death for individuals grappling with these aggressive malignancies [5].

Understanding the patterns and disparities in the place of death for those affected by malignant neoplasms of the meninges is crucial for healthcare professionals, policymakers, and researchers alike. It can inform targeted interventions to improve end-of-life care, enhance patient and family support, and guide resource allocation to better accommodate the unique needs of this patient population [6,7].

Aims and objectives

This research seeks to analyze trends in mortality and end-of-life care preferences via the CDC-WONDER database between the years 1999 and 2020. We also aim to investigate potential disparities in mortality rates and end-of-life care choices based on demographic variables such as age, gender, race, and region, and identify potential factors underlying these differences.

## Materials and methods

Data source

The present epidemiological study utilized data from the CDC-WONDER (Wide-Ranging Online Data for Epidemiologic Research), a publicly accessible resource containing comprehensive public health information, including death certificate data from the United States since 1999. The database is free to use, and the study was deemed exempt from ethical approval due to the use of publicly available, de-identified data. Data extraction was performed on August 31, 2023, focusing on deaths caused by malignant neoplasm of the meninges (ICD-11 code C70).

Cohort selection

The data for this study were gathered from the CDC-WONDER website to include deaths due to malignant neoplasm of the meninges from 1999 to 2020. The study included only records where the underlying cause of death was specified as malignant neoplasm of the meninges, categorized by Bridged-Race Categories. To maintain confidentiality, the analysis adhered to the death count threshold of nine counts.

Death records with a confirmed underlying cause of death as malignant neoplasm of the meninges (ICD-11 code C70) and complete demographic information (age, gender, race, and census region) were included in the analysis.

Deaths were further categorized based on place of death, including home, hospice care, medical facilities, nursing homes, long-term care facilities, unknown places, and others.

Analysis was done by sub-categorizing the data based on age groups (categorized into ten-year intervals from 0 to 85 and above), genders (male and female), race (American Indian, Alaska Native, Asian, Black or African American, and White), and census region of the United States (Northeast, Midwest, South, and West).

Records with incomplete or missing demographic data (e.g., age, gender, or race), that did not specify the underlying cause of death as malignant neoplasm of the meninges, and where the cause of death was secondary to other conditions or diseases, were excluded from the analysis.

Statistical analysis

The data collected were exported to Microsoft Excel (Microsoft Corporation, Redmond, Washington) for further analysis. Statistical analysis was performed using RStudio v4.3.2 (R Foundation, Boston, Massachusetts). An AutoRegressive Integrated Moving Average (ARIMA) model was utilized to forecast trends from 2021 to 2025 based on the gathered data.

Univariate Logistic Regression was applied to identify associations between demographic variables and the place of death, with a p-value < 0.05 considered statistically significant.

## Results

A total of 3068 deaths from 1999 to 2020 were obtained for malignant neoplasm of the meninges from the CDC-WONDER database.

Table 1 displays deaths due to malignant neoplasm of the meninges categorized by the place of death from 1999 to 2020. Based on a 10-year age group analysis, for home or hospice settings, the minimum number of deaths (n=13) occurred in the 15-24 age group, while the maximum (n=346) occurred in the 65-74 age group. In medical or nursing facilities, there were no deaths in the 5-14 and 15-24 age groups, but the maximum (n=385) deaths were observed in the 75-84 age group. For other settings, there were no deaths in four age groups ranging from 5 years to 44 years, while the maximum (n=43) deaths occurred in the 75-84 age group. In terms of gender, females had more deaths than males in all settings, with the highest number of deaths occurring in medical and nursing facilities and the lowest in other settings for both genders. For the Census Region, the highest number of deaths (n=492) was observed in the South region for home or hospice settings, while the lowest (n=16) occurred in the Northeast region for other settings. Based on race, White individuals had the highest number of deaths compared to other racial groups, such as Asian or Pacific Islanders, who had the lowest number of deaths.

**Table 1:** Deaths due to malignant neoplasm of the meninges categorized by the place of death that occurred from 1999 to 2020.

	Home or Hospice (n = 1377)	Medical Facility or Nursing (n = 1519)	Others (n = 172)
10-year age groups			
5-14 years	14	0	0
15-24 years	13	0	0
25-34 years	25	23	0
35-44 years	68	68	0
45-54 years	118	178	17
55-64 years	273	286	30
65-74 years	346	322	37
75-84 years	325	385	43
85+ years	184	208	35
Gender			
Female	708	813	102
Male	669	706	70
Census Region			
Census Region 1: Northeast	215	342	16
Census Region 2: Midwest	288	350	41
Census Region 3: South	492	428	58
Census Region 4: West	382	395	57
Race			
Asian or Pacific Islander	53	83	0
Black or African American	160	212	16
White	1158	1218	150

Table 2 presents predictors of home or hospice deaths due to malignant neoplasm of the meninges from 1999 to 2020. On univariate logistic regression, the reference was set as 1 for the 45-54 age group. Compared to this group, the 35-44 age group (p=0.015) was 1.653 times more likely to experience a home or hospice death. Based on gender, with females as the reference group (1), males had 1.114 times higher likelihood of a home or hospice death. However, this result was not statistically significant, as the p-value was greater than 0.05 (p=0.137). Among Census regions, the Northeast region (Region 1) was taken as the reference (1). Compared to this, Region 3-South (p<0.001) had a 1.686 times higher likelihood of a home or hospice death. For race, the White race was taken as the reference group (1), but the results for both Asian and Pacific Islanders (p=0.118) and Black or African American (p=0.09) groups were not statistically significant, as the p-values were greater than 0.05.

**Table 2:** Predictors of home or hospice deaths due to malignant neoplasm of the meninges from 1999 to 2020.

Variables	Univariate Logistic Regression		
	Odds Ratio	95% Confidence Interval	P-value
Age			
5-14 years	9515029.727	(0, Inf)	0.967
15-24 years	9515029.725	(0, Inf)	0.968
25-34 years	1.796	(0.975, 3.308)	0.06
35-44 years	1.653	(1.101, 2.481)	0.015*
45-54 years	1.0 (Reference)		
55-64 years	1.428	(1.079, 1.889)	0.013*
65-74 years	1.593	(1.213, 2.091)	0.001*
75-84 years	1.255	(0.958, 1.644)	0.1
85+ years	1.251	(0.929, 1.686)	0.141
Gender			
Male	1.114	(0.966, 1.285)	0.137
Female	1.0 (Reference)		
Census Region			
Census Region 1: Northeast	1.000 (reference)		
Census Region 2: Midwest	1.226	(0.977, 1.54)	0.079
Census Region 3: South	1.686	(1.366, 2.081)	<0.001*
Census Region 4: West	1.407	(1.133, 1.749)	0.002*
Race			
Asian or Pacific Islander	0.754	(0.53, 1.074)	0.118
Black or African American	0.829	(0.667, 1.03)	0.09
White	1.000 (reference)		

**Figure 1:**
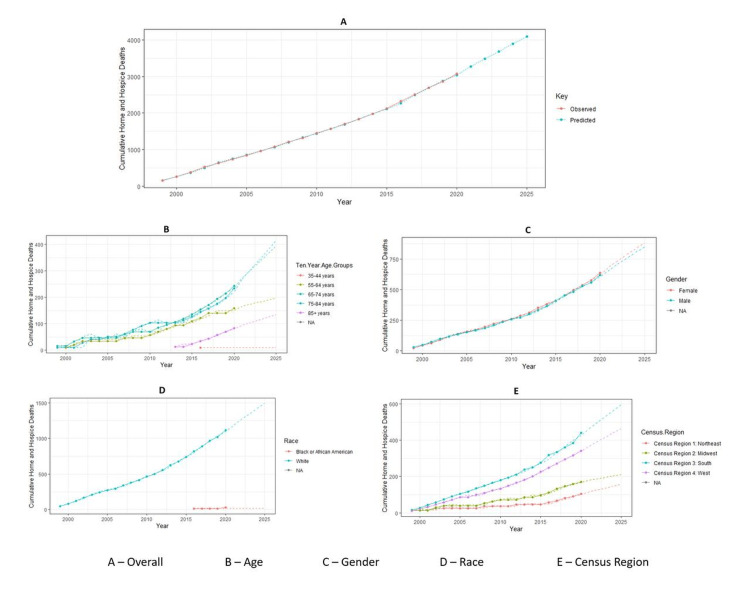
Cumulative trends in home or hospice deaths due to malignant neoplasm of the meninges from 1999 to 2025.

Figure 1 illustrates cumulative trends in home or hospice deaths due to malignant neoplasm of the meninges from 1999 to 2025. The figures were generated using available data from 1999 to 2020, with predictions extending for an additional five years until 2025. Figure 1A displays the overall observed and predicted deaths in home or hospice facilities, showing a gradual increasing trend for both. Figure 1B represents home or hospice deaths by age group, with the highest increasing trends in the 65-74 and 75-84 age groups, and the lowest stationary trend in the 35-44 age group. Figure 1C shows home or hospice deaths by gender, demonstrating a gradual overlapping increasing trend for both males and females. Figure 1D displays home or hospice deaths by race, indicating an increasing trend among White individuals and a stationary trend among Black or African American individuals. Figure 1E represents home or hospice deaths by Census region, with all regions showing increasing trends, most notably in the South (Region 3) and least in the Northeast (Region 1).

## Discussion

To study mortality trends of malignant neoplasm of the meninges, a 22-year dataset was collected from CDC-WONDER. There were 1377 home or hospice deaths, 1519 medical or nursing facility deaths, and 172 deaths in other areas.

Aggregate data of 3068 deaths from 1999 to 2020 showed that, based on age group, the highest number of deaths occurred in the 65-74 years age group for home or hospice care, and the 75-84 years age group for both medical or nursing facilities and other settings. Overall, the highest number of deaths was observed in the 75-84 years age group. This finding aligns with a study conducted by Westwick and Shamji, who used the SEER database to analyze causes of spinal meningioma between 2000 and 2010, showing that all-cause survival was lowest in patients above 80 years of age [8].

Univariate logistic regression in our study revealed that predictors of home or hospice deaths were more likely in the 35-44 years age group, even though total deaths were more common among the 75-84 years age group. This contrasts with findings by Talha et al., who observed that younger patients with congenital heart disease were more likely to die in hospitals, while older patients were more likely to die in home or hospice settings [9]. The difference in findings could be attributed to younger patients experiencing different trajectories of disease progression, leading to different end-of-life care decisions compared to older patients [10].

Our study observed that overall deaths were higher among females in home or hospice care, medical or nursing facilities, and other settings. Males were more likely to have hospice or home deaths compared to females, but this result was statistically insignificant. However, other studies have found significant associations between male gender and mortality rates. For example, the study by Westwick and Shamji indicated that the Cox hazard function for overall mortality in males was higher (2.4 (95% CI 1.7-3.5)) [8]. Another study by Cao et al., using the SEER database on the epidemiology and survival of patients with spinal meningiomas, also found that females had better overall survival rates than their male counterparts [11]. Furthermore, Dolecek et al., in a study on the epidemiology of meningiomas post Public Law 107-206, found poorer survival outcomes among male patients, despite lower incidence rates compared to females [4].

In the present study, the highest number of home or hospice deaths occurred in the South region, while the lowest was in the Northeast. This can be attributed to regional differences in healthcare accessibility and preferences, economic factors, demographic variations, availability and quality of hospice services, physician practices, state policies, and cultural norms regarding end-of-life care [12-15].

The aggregate data show that White individuals had the highest number of home or hospice deaths, medical or nursing facility deaths, and deaths in other areas. The present study also found that death rates among Asian and Black individuals were lower than those of White individuals, though this was statistically insignificant. In contrast, studies by Dolecek et al. and Achey et al. show that death rates were significantly higher among Black patients [4, 11]. Thus, further studies on these discrepancies are warranted.

Limitations

This study has several limitations that should be acknowledged. First, the analysis did not include the most recent data from 2021 to 2023, which may affect the completeness and relevance of the findings. As medical trends, treatment modalities, and healthcare practices evolve, the exclusion of these recent data might limit the applicability of the results to current clinical settings. Secondly, the classification of malignant neoplasm of the meninges was not further subdivided into specific histological subtypes or stages of the disease. This lack of detailed categorization may obscure important variations in prognosis, treatment outcomes, and patient management strategies, potentially affecting the study's ability to capture the full spectrum of the disease.

Moreover, as an epidemiological study, our analysis is limited in its ability to make inferences about prognostic factors or the underlying reasons for death. The absence of data on treatment regimens, comorbidities, and disease stage significantly constrains our capacity to explore how these factors might influence mortality and end-of-life care decisions. Consequently, while our findings shed light on demographic trends and disparities, they do not provide a comprehensive understanding of the clinical and social determinants that contribute to mortality in patients with malignant neoplasm of the meninges.

Despite these limitations, the study offers valuable insights into the demographic factors associated with malignant neoplasm of the meninges and highlights the critical intersection between this condition and end-of-life care. Future research should focus on incorporating detailed clinical data, including treatment histories, comorbidities, and disease staging, to better understand the prognostic factors influencing patient outcomes. Additionally, longitudinal studies that capture evolving trends in healthcare practices and patient management could provide a more nuanced understanding of how these variables impact mortality and end-of-life care decisions. Exploring these areas will be essential for developing targeted interventions and improving care for patients with malignant neoplasm of the meninges.

## Conclusions

Our analysis revealed distinct age-related trends, highlighting the disproportionate impact of this condition on the elderly population, particularly those in the 65-74 and 75-84 age groups. However, it is noteworthy that the 35-44 age group exhibited a higher likelihood of home or hospice deaths, emphasizing the nuanced nature of end-of-life care preferences among different age cohorts. Furthermore, gender disparities were evident, with females consistently experiencing higher mortality rates, while males showed a slightly higher likelihood of home or hospice deaths. Geographically, our study highlighted regional variations in end-of-life care, with the South region recording the highest numbers of home or hospice deaths, contrasting with the lowest occurrences in the Northeast. Additionally, we identified racial disparities in mortality rates, with White individuals experiencing higher death rates than Asian or Pacific Islanders and Black or African American individuals. While our findings highlight important demographic trends and disparities, we acknowledge that further research incorporating additional clinical data is essential to fully understand the underlying factors contributing to these differences.

To improve end-of-life care for patients with malignant neoplasm of meninges, several measures should be considered. First, healthcare providers should prioritize tailored care plans that align with the unique needs and preferences of patients in different age groups, acknowledging the evolving dynamics of end-of-life decision-making. Secondly, strategies to reduce gender disparities in care choices should be explored, with a focus on enhancing access to information and support for both male and female patients. Moreover, addressing regional variations in end-of-life care may necessitate targeted interventions, including the development of healthcare infrastructure and culturally sensitive programs in regions with lower home or hospice death rates. By recognizing the multifaceted nature of this issue and implementing future measures to enhance care quality and equity, we can aspire to provide more compassionate and patient-centered end-of-life care for individuals facing this challenging medical condition and their families.
